# Development and Validation of the General Dietary Behavior Inventory (GDBI) in Scope of International Nutrition Guidelines

**DOI:** 10.3390/nu13041328

**Published:** 2021-04-17

**Authors:** Gerrit Engelmann, Matthias Marsall, Eva-Maria Skoda, Nadja Knoll-Pientka, Laura Bäuerle, Nanette Stroebele-Benschop, Martin Teufel, Alexander Bäuerle

**Affiliations:** 1 Clinic of Psychosomatic Medicine and Psychotherapy, University of Duisburg-Essen, University Hospital, 45147 Essen, Germany; matthias.marsall@stud.uni-due.de (M.M.); eva-maria.skoda@uni-due.de (E.-M.S.); nadja.knoll-pientka@uni-due.de (N.K.-P.); martin.teufel@uni-due.de (M.T.); alexander.baeuerle@uni-due.de (A.B.); 2 Hochschule Niederrhein, Faculty of Food, University of Applied Science, Nutrition and Hospitality Science, 47805 Krefeld, Germany; laura.baeuerle@hs-niederrhein.de; 3 Institute of Nutritional Medicine, University Hohenheim, 70599 Hohenheim, Germany; n.stroebele@uni-hohenheim.de

**Keywords:** dietary behavior, inventory, dietary recommendation, weight, Body Mass Index, construct validation, criterion validity, cluster analysis

## Abstract

Unhealthy eating is associated with various diseases, such as cardiovascular, neurodegenerative, or oncological. There are neither economical nor behavior-related questionnaires available in the German language to assess general dietary behavior. Therefore, the aim of this validation study was to develop an instrument considering these aspects and verifying its construct and criterion validity. The new questionnaire is based on the general nutrition recommendations of the World Health Organization and the German Nutrition Society. It consists of 16 items that contrast dietary behaviors on a semantic differential scale. Our German-speaking convenience sample consisted of 428 participants. The construct validity of the General Dietary Behavior Inventory (GDBI) could be confirmed by examining convergent and discriminant validity. Furthermore, criterion validity was confirmed (significant negative correlations with body weight, Body Mass Index, and positive correlations with physical/mental health as well as life satisfaction). A cluster analysis revealed two different dietary behavior clusters representing a rather healthy and a rather unhealthy dietary behavior cluster. The results indicate that the GDBI is a validated and economical instrument to assess general dietary behavior. In practical research, this questionnaire helps to assess dietary behavior and to derive interventions for a healthy dietary behavior in concordance with international nutrition recommendations.

## 1. Introduction

In empirical research, dietary behavior is described as an important variable related to one’s health status and body weight [1,2]. A large number of studies and reviews have shown correlations between dietary behavior and diseases such as cardiovascular, metabolic (e.g., diabetes mellitus), neurodegenerative, or oncological [3,4,5,6]. Likewise, a relationship between unhealthy or stress-induced dietary behaviors with obesity has been demonstrated [7,8]. Furthermore, eating disorders, which are related to unbeneficial dietary behaviors, point to long-term negative physical as well as psychological health consequences or behaviors such as under- or overweight, smoking, higher mortality risk, depression, or anxiety disorders [9,10,11]. Therefore, the measurement of dietary behavior is an important indicator of one’s physical and mental health. To assess dietary behavior, self-report questionnaires are mostly used, which focus on different aspects of dietary behavior, such as:

(1) Food frequency questionnaires [12,13] that focus on food, nutrient, or energy intake rather than on specific dietary behavior, so that these questionnaires measure dietary intake rather than general dietary behavior [14];
(2) Questionnaires to assess the psychological components of dietary behavior measure attitudes toward diet, eating habits, or individual cognitions or emotions influencing dietary behavior [15,16,17,18];
(3) Questionnaires for the assessment of disorder-specific eating behavior or weight problems [19,20,21] focusing on specific maladaptive changes in dietary behavior due to psychological disorders [22]. Each of these questionnaires focuses on different aspects of disordered eating behavior and does not assess general dietary behavior;
(4) Questionnaires for the qualitative assessment of dietary behavior evaluating specific dietary recommendations, such as healthy eating indexes [23,24,25].

On closer examination, food frequency questionnaires mostly focus on energy intake and do not capture specific dietary behaviors [14]. Furthermore, these questionnaires usually assess portion sizes and amounts of foods, which are hard to remember and to evaluate retrospectively by respondents. Due to this, measuring dietary behavior by food frequency questionnaires results in methodological inaccuracies such as biased means or variances [26]. In addition, because of the high number of items in most of these questionnaires [13,27,28], food frequency questionnaires are uneconomical tools for assessing dietary behavior. Questionnaires assessing the psychological components of dietary behavior do not evaluate dietary quality or quantity and in this case only refer to the psychological aspects influencing dietary behavior [17,18,29]. Furthermore, questionnaires for the assessment of disorder-specific eating behavior or weight problems do not focus on general dietary behavior but on disordered eating behavior. Questionnaires for the qualitative assessment of dietary behavior are mainly based on food frequency questionnaires resulting in similar methodological problems.

To sum up, all questionnaire formats hardly capture concrete dietary behavior. The items barely describe self-determined behavioral situations and thus only represent abstract situations or hard-to-remember food-related aspects for subjects. In addition, questionnaires for the qualitative assessment of dietary behavior only assess dietary behavior based on specific dietary recommendations that only refer to energy intake or dietary quality [23,24].

Moreover, none of the questionnaires look at dietary behavior in its entirety. Dietary behavior itself consists of different aspects: Healthy eating does not only focus on food or its energy and nutrients but also includes food handling such as preparation of a meal and the way food is eaten. Food handling and the intake of meals are associated with different outcomes of dietary behavior [30,31]. A higher likelihood of time spent in meal preparation is especially related to a healthy diet [31]. Reviews and meta-analyses have shown that mindful eating is associated with weight loss [30,32]. In addition to energy or nutrient intake, the abovementioned aspects of a healthy diet should be considered to evaluate dietary behavior in its entirety. To the best of our knowledge, there is no questionnaire considering dietary behavior with its different aspects to assess this complex behavior. An assessment of all these mentioned aspects is still missing in practical and scientific research.

Furthermore, it is important to look at the criterion validity of existing questionnaires. Criterion validity of a questionnaire describes the ability of a questionnaire to predict relationships between the construct itself and a possible outcome [33] and is therefore an important indicator of the validity of the instrument. Criterion validity of different dietary assessments has been examined and showed associations with health-related outcomes like cardiovascular disease [34], Body Mass Index (BMI) [35], or amount of food intake [18]. Questionnaires capturing psychological aspects of dietary behavior are associated with weight- or health-related dietary behaviors [2,36,37]. However, different authors describe the major problem of self-reported dietary records (e.g., food frequency questionnaires) by revealing a distorted perception of one’s food consumption retrospectively, resulting in unreliable estimation of outcomes such as energy intake [38,39].

In conclusion, existing questionnaires measuring dietary behavior show methodological deficiencies such as biased means or variances or unreliable estimation of outcomes. Additionally, content-related deficiencies are present in existing questionnaires, and questionnaires often only measure specific aspects of dietary behavior or focus on divergent dietary behavior. Moreover, food frequency questionnaires with a high number of items are uneconomical assessment tools for dietary behavior. Thus, it becomes evident that there is a need for further development in this field. To not only assess dietary behavior based on food, energy or nutrient intake or psychological components, it is necessary to look at dietary behavior in its entirety by examining general dietary behavior on behalf of different aspects. Here, it is important to include aspects such as food handling and meal intake. In this context, we developed a new instrument for measuring dietary behavior aiming at several objectives: First, we developed a questionnaire based on recommendations of the World Health Organization (WHO) and the German Nutrition Society (DGE) [40,41]. Second, our questionnaire considers self-determined situations to assess concrete typical dietary behaviors and is not biased by retrospective exceptional situations such as current diets or past occasions resulting in a special diet. Third, we wanted to assess entire dietary behavior by considering relevant behaviors associated with dietary behavior. Fourth, it was our goal to develop a questionnaire which measures dietary behavior as economically as possible and to scrutinize convergent and discriminant validity to confirm construct validity. Additionally, we wanted to ensure the criterion validity of the newly developed questionnaire. Fifth, we wanted to verify whether the newly developed questionnaire could distinguish between healthy and unhealthy dietary behavior clusters according to international nutrition recommendations.

## 2. Materials and Methods

### 2.1. Participants and Study Design

A cross-sectional online survey was conducted in a German-speaking convenience sample. Participants for this study were recruited via online social networks (e.g., Xing, Facebook). In our analyses, only complete data sets were considered. We excluded cases in which participants took less than 7:32 min (5% percentile) or more than 30:37 min (95% percentile) to complete the survey. Furthermore, we excluded two participants for being under 18 years as well as three participants due to false responses in body weight or height. The resulting sample consisted of *N* = 428 persons. Answering the questionnaire took approximately 13:54 min on average (SD = 4:53 min). Completion rate was 43.2%.

The survey was conducted via Unipark (Questback GmbH), an online survey tool, between December 2020 and January 2021. To avoid bias due to change in dietary behavior, we paused the data collection in the period between 20 December and 13 January. All participants gave electronic informed consent to the study conditions before participating in the study. The study was conducted according to the guidelines of the Declaration of Helsinki and approved by the Ethics Committee of the medical faculty of the University of Duisburg-Essen (approval number 20-9718-BO).

### 2.2. Development of the General Dietary Behavior Inventory

The developed “General Dietary Behavior Inventory” (GDBI) for the assessment of general dietary behavior is intended to provide an answer to the deficits in terms of content and methodology in the assessment of dietary behavior. The new instrument is based on general nutrition recommendations of the WHO [41,42] and the DGE [40,43,44], which have formulated easy-to-understand recommendations for general nutrition. From these recommendations, indications for a health-related dietary behavior can be concluded. We developed a 20-item questionnaire based on the above-mentioned recommendations. The questionnaire was tested for content validity by two independent nutritionists, resulting in further adjustments. We developed a 5-point bipolar scale, oriented to the semantic differential [45], that depicts concrete opposite dietary behaviors. Participants had to choose between these opposite dietary behaviors reflecting most closely their typical, everyday dietary behavior. A sample item is “I do not eat sweets (e.g., chocolate, cookies, pastries)” compared to “I eat sweets (e.g., chocolate, cookies, pastries) every day”. The final version of the original and translated questionnaire is displayed in Table S1. The scoring of each item was from 1 (=Like behavior A) to 5 (=Like behavior B). We inverted several items to prevent assessment against response tendencies of participants.

### 2.3. Study Variables

In the study, it was our objective to validate the GDBI in a convenience sample, to verify its convergent, discriminant, and criterion validity. Convergent validity assumes that measurements representing similar constructs are significantly correlated, whereas discriminant validity assumes that measurements representing different constructs are not significantly correlated [46]. We verified convergent validity by assuming a positive correlation between GDBI sum score and attitude toward food. Furthermore, we expected a positive interrelation with nutrition knowledge. To verify discriminant validity, we captured interpersonal trust as well as a belief about a just world. Referring to the work of Campbell and Fiske [46], discriminant validity can be assumed when constructs have no content-related overlap and do not correlate significantly. Different studies and meta-analyses have shown intercorrelations between eating disorders/dietary behavior and different personality constructs, e.g., [47,48], or emotional states [49]. Thus, it was our objective to identify constructs having a low agreement with dietary behavior. To test the discriminant validity of the GDBI, we used the constructs of interpersonal trust and the general just world as they show no content-related overlap but capture daily behavioral aspects and values of individuals. Additionally, criterion validity was assessed by investigating interrelations between the GDBI and body weight, BMI, life satisfaction, and physical and mental health.

#### 2.3.1. Attitude toward Food

The subdimension “attitude toward food” from the Eating Behavior and Weight Problems Inventory [50] measures with eight items the significance and relevance of food. A sample item is “Food is one of the best parts of life”. The participants answer the statements on a 4-point Likert scale from 1 = “does not apply to me” to 5 = “applies to me” (M = 3.22 ± 0.54). Higher scores on the scale indicate a higher importance of food and its intake. The Cronbach’s alpha of this scale was 0.89.

#### 2.3.2. Nutrition Knowledge

The nutrition knowledge questionnaire [51] measures general nutrition knowledge on a 20-item scale. Items are measured on a 3-point scale (right/wrong or do not know). Therefore, it was used as a sum score which indicated the extent of nutrition knowledge between 0 and 20 (M = 14.92 ± 3.08). A sample item is “Bacon contains more calories than ham”. A higher score indicates greater nutrition knowledge. The Cronbach’s alpha of this scale was 0.66.

#### 2.3.3. Interpersonal Trust

The interpersonal trust questionnaire [52] measures the experiences of interpersonal trust by three items. The participants answered the statements on a 5-point Likert scale from 1 = “strongly disagree” to 5 = “strongly agree” (M = 3.37 ± 0.87). A sample item is “In general, people can be trusted”. A higher score on this scale portrays a higher trust in interpersonal relationships. The Cronbach’s alpha of this scale was 0.81.

#### 2.3.4. General Just World

The general just world scale [53] measures the general belief in a just world with six items. The participants answer the statements on a 4-point Likert scale from 1 = “not true at all” to 4 = “true at all” (M = 2.58 ± 0.84). A sample item is “I think the world is generally fair”. A higher score indicates a greater trust in a fair or just world. The Cronbach’s alpha of this scale was 0.78.

#### 2.3.5. Further Constructs and Sociodemographic Variables

Additionally, single items were used to measure life satisfaction on a 5-point Likert scale from 1 = “not satisfied at all” to 5 = “totally satisfied” (mean = 3.7 ± 0.92) [54], and physical (mean = 7.48 ± 1.72) as well as mental health (mean = 7.17 ± 2.03) on a 10-point Likert scale from 0 = “very bad health” to 10 = “very good health” (self-formulated). A greater score on these items implied a greater satisfaction in life and a higher physical and mental health status. Sociodemographic variables (age, gender, marital status, educational degree, body weight (in kilogram) and body height (in meters)) were assessed. Further, we asked participants about their general diet (1 = omnivore diet, 2 = vegetarian diet, 3 = vegan diet, 4 = other diet) and food allergies or intolerances.

### 2.4. Statistical Analysis

In this study, it was our objective to validate the GDBI on a convenience sample by verifying its convergent, discriminant and criterion validity. Descriptive statistics were calculated, and data analyses were considered significant at *p* < 0.05.

To distinguish between healthy and unhealthy dietary behavior clusters, we carried out a k-means cluster analysis with the means of the GDBI items. We determined the optimal number of clusters based on the elbow method and validated the results by considering the silhouette coefficient [55]. For examination of silhouette coefficients, data were z standardized.

All data analyses were conducted using R [56] and RStudio [57]. Data analysis was carried out using the packages psych [58], semtools [59], hmisc [60], and factoextra [61]. These tools were used to identify correlations between the collected constructs and to test criterion validity. To test for validity, we performed analyses of Pearson correlations. Criterion validity was examined by relations to body-related and psychological variables which are generally associated with dietary behavior.

## 3. Results

### 3.1. Sample Characteristics

The mean age of the participants was 34.8 ± 11.1 years. In total, 317 of the participants were female, 109 were male, and 2 indicated their gender as diverse. Of our participants, 305 were married or in a partnership and 123 indicated being single or “other”. The educational background of our participants was assessed by a seven-point scale from 1 = “no school leaving certificate” to 7 = “University degree (of applied sciences)”. In sum, 234 persons held a university degree (of applied sciences), 82 had completed a vocational training, which is a German-specific practical training of 2–3 years with a final graduation, and 111 had a school degree. One person was without a school leaving certificate. The mean body weight of the participants was 79.3 ± 23.3 kg, and mean height was 1.72 ± 0.09 m. The resulting mean BMI was 26.8 ± 7.8 kg/m^2^. Mean body weight of women was 76.6 ± 24.7 kg, and mean height was 1.69 ± 0.07 m. The resulting mean BMI of women was 26.9 ± 8.7 kg/m^2^. The mean body weight of men was 87.5 ± 16 kg, and mean height was 1.82 ± 0.07 m. The resulting mean BMI of men was 26.4 ± 4.5 kg/m^2^. A total of 18 participants were underweight (BMI < 18.5 kg/m^2^), 222 participants were normal weight (18.5 kg/m^2^ ≤ BMI < 25 kg/m^2^), 85 participants were overweight (25 kg/m^2^ ≤ BMI < 30 kg/m^2^), and 103 participants were obese (BMI ≥ 30 kg/m^2^). To compare the distribution of BMI in our sample to country-specific BMI, an age- and gender-corrected standard deviation score of BMI was calculated [62]. In our sample, women differed from the age-corrected median BMI by 0.26 on average. Men’s BMI was 0.08 higher than age-corrected median BMI on average. In sum, age- and gender-corrected BMI differed by 0.22, indicating that our sample represents the country-specific distribution concerning BMI. In total, 296 participants indicated eating an omnivorous diet, 54 a vegetarian diet, 47 a vegan diet, and 31 other diets. In total, 82% of participants stated not having any food allergy or intolerance.

### 3.2. Descriptive Statistics of the General Dietary Behavior Inventory

The GDBI consisted of 20 items. Four questionnaire items were excluded due to methodological and content-related reasons: one item did not reflect a typical dietary behavior but the degree of physical activity, which is a recommendation of both societies but not related to dietary behavior. Three items were excluded due to high content-related overlap with other items. The item statistics of the final 16-item questionnaire are shown in Table 1. Looking at the skewness of the items, most of them are negative, indicating a slightly left-skewed distribution of these items. A sum score over the 16 items was calculated. The GDBI sum score was derived from the respective mean value, resulting in a score between 16 and 80. In our sample, the lowest score was 36, and the highest score was 80 (M = 56.71 ± 7.9, Med = 57). A higher score indicated a healthier dietary behavior in the scope of the recommendations of the WHO and the DGE. We calculated a sum score due to methodological reasons, since dietary behavior is basically dependent on many different components, which, however, are not necessarily related to each other. As an example, the consumption of sweets, vegetables, or animal products, etc., does not necessarily have to correlate with, but does represent, the latent variable of dietary behavior in definitional terms. Looking at the intercorrelations of dietary behavior items (Table S2), this assumption could be confirmed. The dietary behavior items are partly correlated. Thus, convergent and discriminant validity tests were performed between the sum score of the GDBI and other scales that are close to or distant from the GDBI. The Cronbach’s alpha of this scale was 0.73. However, Cronbach’s alpha can only be interpreted to a limited extent because of the above-mentioned construct variety of dietary behavior.

### 3.3. Relationships between Dietary Behavior and Sociodemographic Variables

As a first step, correlations between dietary behavior and sociodemographic variables were conducted. As only two participants indicated their gender as diverse, they were not considered in the following gender-comparing analyses. Men and women differed significantly in body weight (*t* = −4.28, *p* < 0.001), but not in BMI (*t* = 0.61, *p* = 0.54). There were no significant differences between gender in the GDBI score (*t* = 1.28, *p* = 0.20), attitude toward food (*t* = −0.63, *p* = 0.53), or nutrition knowledge (*t* = 1.74, *p* = 0.08).

Looking at the correlation between the GDBI score and general diet, there was a significant correlation (*r* = 0.31, *p* < 0.001), but neither with sociodemographic variables nor with food allergies (Table 2). In total, 31 persons with other diets had to be excluded from the correlation between GDBI score and general diet because their diet could not be specified, and results would indicate inaccuracies.

### 3.4. Validity Test and Cluster Analysis

The GDBI was significantly related to the scales “attitude toward food” (*r* = 0.14, *p* < 0.01) and “nutrition knowledge” (*r* = 0.18, *p* < 0.001), confirming convergent validity (Table 3). No correlation was found between the GDBI and the scales “interpersonal trust” (*r* = 0.04, *p* = 0.42) and “general just world” (*r* = −0.02, *p* = 0.65), indicating discriminant validity. Significant relationships were found between the GDBI and body weight (*r* = −0.15, *p* < 0.01), BMI (*r* = −0.13, *p* < 0.01), physical health (*r* = 0.27, *p* < 0.001), mental health (*r* = 0.13, *p* < 0.01), and life satisfaction (*r* = 0.16, *p* < 0.001), confirming criterion validity of the newly developed GDBI.

**Figure 1:**
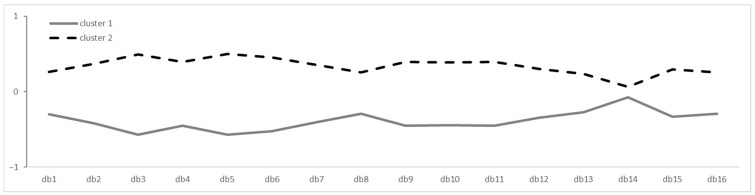
Cluster distribution over 16 GDBI items. Graph of K-clustering means results over 16 GDBI items on a z-standardized scale (*n* = 428).

In order to determine the appropriate number of clusters, the 16 items of the GDBI were considered in an elbow analysis indicating the elbow point at two or three clusters. Results revealed that two clusters had a higher silhouette coefficient (mean = 0.13) than three clusters (mean = 0.10). Subsequently, participants were assigned to one of the two clusters. Figure 1 shows how clusters distribute over the 16 GDBI items.

Cluster assignments were correlated with scales of criterion validity. The results are equivalent to the correlations of the GDBI score and the scales of criterion validity. Significant relationships were found between the GDBI cluster assignment and body weight (*r* = −0.21, *p* < 0.001), BMI (*r* = −0.21, *p* < 0.001), physical health (*r* = 0.29, *p* < 0.001), mental health (*r* = 0.10, *p* < 0.05), and life satisfaction (*r* = 0.15, *p* < 0.01).

## 4. Discussion

The first aim of our study was to develop a new instrument to assess dietary behavior based on general nutrition recommendations of the WHO [41,42] and the DGE [40,43,44]. Our second and third aim was to design an economical and behavior-related questionnaire capturing self-determined situations available in the German language to assess actual dietary behavior in its entirety. We achieved these goals by validating the newly developed General Dietary Behavior Inventory (GDBI). According to the fourth aim of our study, we examined convergent and discriminant validity by looking at measurements representing similar and different constructs to dietary behavior. Thus, we examined content validity by two independent nutritionists. The criterion validity of our new questionnaire was also confirmed by investigating correlations with important outcomes of dietary behavior. Our fifth aim contributed to the distinction between healthy and unhealthy dietary behavior clusters according to the above-mentioned recommendations. We looked at resulting dietary behavior clusters in our newly developed questionnaire.

Results indicated that the validity of the GDBI can be assumed. As expected, the GDBI correlated significantly with the convergent constructs of nutrition knowledge and attitude toward food. This indicates that a greater score in the GDBI is associated with greater nutrition knowledge and a more positive attitude toward food. Thus, a higher GDBI score is associated with a higher subjective importance of food and its intake. Furthermore, it could be confirmed that the GDBI did not interrelate to constructs which are different from dietary behavior (interpersonal trust and general just world), confirming discriminant validity. To examine criterion validity, we investigated interrelations with the body-related outcomes body weight, BMI, physical health, and the psychological outcomes mental health and life satisfaction. The results imply that the GDBI is a valid measurement for the prediction of body (weight and BMI) and health-related (physical and mental health, life satisfaction) outcomes. As the WHO describes “health positively, as complete physical, mental and social well-being, not merely negatively as the absence of disease or infirmity” [63], these outcome variables are important indicators to describe one’s overall health status. Thus, the external validity of the GDBI can be assumed, and a higher score in the GDBI indicates a healthier dietary behavior in the scope of the recommendations of the WHO and DGE. Analyses of dietary behavior and sociodemographic variables showed no significant interrelations. General diet was associated with the GDBI, indicating that the GDBI score is positively related to a vegan or vegetarian diet. This can be explained as dietary behavior following a vegan or vegetarian diet is more in line with the nutrition guidelines of the WHO [41,42] and the DGE [40,43,44], which suggest a plant-based diet except for eating moderate amounts of meat or fish. Here, it is important to take the average meat consumption per capita into account because in western civilization, there is a high meat consumption going beyond the recommendations of nutrition guidelines [64]. This suggests that people in these societies consistently exceed the recommended amounts of meat and therefore do not follow the recommendations of the WHO and DGE. Taking this into account, the association between GDBI score and general diet can be explained.

It was found that the GDBI score was negatively associated with individuals’ BMI and body weight. Obesity and BMI are relevant indicators of different health risks [65,66], cardiovascular diseases, metabolic, or oncological diseases [67,68]. Participants did not enter their BMI themselves, but it was calculated based on their data (height and body weight). Thus, we could avoid bias because of incorrect BMI calculation or unawareness of one’s own BMI. Moreover, it could be demonstrated that following the recommendations is significantly positively associated with physical and mental health as well as life satisfaction. Overall, the results show that the GDBI is a useful instrument to reveal the associations of general dietary behavior and health-related outcomes. In contrast to other self-report questionnaires measuring dietary behavior, which do not correlate negatively with BMI [36], the GDBI has methodological strengths and has advantages over other dietary behavior questionnaires. The association of dietary behavior and life satisfaction has already been identified in several studies supporting our findings [69,70,71]. However, the GDBI did not capture actual food intake in contrast to typical food frequency questionnaires [12,13]. This is because our questionnaire should not assess exact energy intake but different aspects of actual dietary behavior on a typical participant’s day. Specific eating patterns, energy deficiencies, or excessive energy intake cannot be observed because of missing information on energy intake. The results of the GDBI contribute to nutrition research insofar as statements can be made about how different foods are consumed in general. The consumption of convenience products, animal fats, salty foods, and sweets is receiving a great deal of attention because of its relation to diet-related diseases [72,73,74]. Thus, the GDBI could be used as an instrument to estimate the general consumption of unhealthy foods in the population to emphasize the need for strategies and initiatives for a healthy diet in different countries [74]. Additionally, our newly developed dietary behavior inventory refers to the Joint Programming Initiative “A Healthy Diet for a Healthy Life” (JPI-HDHL) funded by the European Union [75] and is in line with current international projects in dietary research [76]. This initiative formulates and supports research projects developing improved methods to assess dietary behavior, and our questionnaire seems to extend as well as supplement survey methods in research of dietary behavior.

Cluster analysis of the GDBI identified two clusters which are distant from each other and revealed two different dietary behaviors indicating a rather healthy and a rather unhealthy dietary behavior according to the recommendations of the WHO and DGE. Looking closely at Figure 1, cluster 2 has a better diet than cluster 1. Furthermore, the greatest difference between those clusters is shown on items number 3 and 5 measuring the consumption of plant-based products and vegetables. Looking at the statistics of cluster analysis, the two-cluster solution sufficiently fits the dataset compared to different multi-cluster solutions. However, considering the value of the silhouette coefficient, it could be assumed that the two clusters are not sufficiently distant from another. Literature showed that silhouette coefficient value should be close to 1, indicating that the set is well clustered [55,77].

Nevertheless, there are limitations in our study that should be considered. One limitation of our study is referring to the methodological design of the questionnaire: We confirmed convergent und discriminant validity by comparing the GDBI with similar and different constructs to dietary behavior, respectively. As dietary behavior reflects very different, uncorrelated behaviors, examination of factorial structure is not common in this scientific field [12,13,78,79]. Dietary behavior depends on different components, which, however, cannot or do not necessarily have to be related to each other. Another limitation refers to the measurement of dietary behavior in our inventory. We developed a questionnaire assuming a constant dietary behavior over a period. Participants had to choose between different dietary behaviors on a bipolar scale representing their typical everyday dietary behavior. However, one can assume that dietary behavior is not a constant behavior but differs over time and depends on individual aspects (e.g., mood or changing food preferences) [80,81,82,83] or circumstances influencing dietary behavior, e.g., the COVID-19-pandemic [84,85]. We cannot exclude selection bias in our study because we conducted an online survey. Thus, the results are probably not generalizable to the overall population. A further limitation of our study refers to the operationalization of meat consumption. The GDBI does not distinguish between different meat products (e.g., processed meats, red meat, lean meat). However, these differences between meat products and their effects on health status are discussed in literature regarding meat consumption [86,87,88].

Conversely, we want to highlight five important strengths of this study. First, we developed a dietary behavior questionnaire based on a deductive procedure and on theoretical reliable recommendations of the WHO and DGE. Second, we developed a questionnaire assessing relevant aspects of dietary behavior going beyond mere food or nutrient intake. To our knowledge, this is the first instrument measuring dietary behavior in its entirety by additionally looking at different aspects such as food handling and the intake of meals. Third, the evaluation of actual dietary behavior based on general nutrition recommendations resulting in a sum score is practical and easy to use in healthcare and science. This helps diagnosticians and practitioners to discuss results with participants and to derive interventions for a healthy diet in line with general recommendations for nutrition. Low scores indicate less compliance with general nutrition recommendations, and results can be used to look at specific dietary behavior patterns and the development of a healthier diet in concordance with the recommendations. Here, we identified two different dietary behavior clusters indicating a rather healthy and a rather unhealthy dietary behavior in line with the recommendations of both associations. Moreover, the GDBI might be a useful instrument to show changes in general dietary behavior over time in longitudinal intervention studies. In our sample, only a few participants were underweight, revealing a need for future studies to validate our questionnaire in samples of underweight participants—with or without specific eating disorders (e.g., orthorexia or anorexia nervosa)—and to detect unhealthy dietary patterns in such clinical samples. Fourth, based on the relationship of the GDBI with low BMI and high physical health, it might be a useful instrument in the therapy of overweight and obese persons because it offers opportunities for possible interventions regarding dietary behavior to reduce BMI. A fifth strength of the developed and validated instrument is the disorder-unspecific acquisition of dietary behavior based on general and behavioral recommendations regarding nutrition, which accounts for the wide range of applications of the GDBI.

## 5. Conclusions

Our findings suggest that the newly developed GDBI is instrumental in economizing and simplifying the assessment of dietary behavior in a large online sample. The construct and criterion validity of this new instrument could be confirmed and indicated adequate psychometric properties. Therefore, the GDBI is applicable in research and clinical practice to assess dietary behavior and to evaluate the results on a basis of general nutrition recommendations. Furthermore, the cluster analysis revealed different dietary behavior clusters, showing that general dietary behavior in line with international nutrition recommendations is associated with positive outcomes of health status such as BMI, physical and mental health, and life satisfaction. Future studies should consider longitudinal data with the GDBI to test the prognostic validity and sensitivity to change of this instrument.

## Figures and Tables

**Table 1:** Item statistics of General Dietary Behavior Inventory (GDBI) items (inverted items were recoded).

Item	Mean	SD	Skew
db1	3.67	0.96	−0.34
db2	3.80	1.19	−0.65
db3	4.24	0.88	−0.99
db4	3.49	1.09	−0.18
db5	3.61	0.91	−0.29
db6	3.31	1.09	−0.12
db7	3.89	1.02	−0.57
db8	2.57	1.15	0.26
db9	3.85	0.81	−0.32
db10	3.76	1.24	−0.81
db11	4.12	1.22	−1.23
db12	3.22	0.95	−0.12
db13	3.03	1.24	0.06
db14	3.72	1.34	−0.64
db15	2.87	1.31	0.23
db16	3.56	1.14	−0.38
GDBI score	56.71	7.90	0.04

**Table 2:** Pearson correlation coefficients of GDBI score and sociodemographic variables, general diet, and food allergies (*n* = 428).

	GDBI	Age	Marital Status	Education Level	General Diet
age	0.0				
marital status	0.05	−0.30 ***			
education level	0.05	−0.17 ***	0.04		
general diet ^1^	0.31 ***	−0.22 ***	0.09	0.09	
food allergies	−0.02	−0.04	0.06	−0.06	0.11 *

* *p* ≤ 0.05, *** *p* < 0.001, ^1^ statistical analysis with *n* = 397.

**Table 3:** Test for construct and criterion validity of GDBI score (*n* = 428).

Scales		GDBI
convergent validity		
	attitude toward food	0.14 **
	nutrition knowledge	0.18 ***
discriminant validity		
	interpersonal trust	0.04
	general just world	−0.02
criterion validity		
	body weight	−0.15 **
	Body Mass Index	−0.13 **
	physical health	0.27 ***
	mental health	0.13 **
	life satisfaction	0.16 ***

** *p* < 0.01, *** *p* < 0.001.

## Data Availability

The data presented in this study are openly available in FigShare, Dataset: doi.org/10.6084/m9.figshare.14055389. R-Syntax: doi.org/10.6084/m9.figshare.14055389.

## Supplementary Materials

The following are available online at https://www.mdpi.com/article/10.3390/nu13041328/s1, Table S1: Original and translated items of the General Dietary Behavior Inventory; Table S2: Table 2. Intercorrelations of General Dietary Behvior Inventory items.

## References

[1] Lake A.A., Hyland R.M., Rugg-Gunn A.J., Wood C.E., Mathers J.C., Adamson A.J. (2007). Healthy Eating: Perceptions and Practice (the ASH30 Study). Appetite.

[2] Wilson-Barlow L., Hollins T.R., Clopton J.R. (2014). Construction and Validation of the Healthy Eating and Weight Self-Efficacy (HEWSE) Scale. Eat. Behav..

[3] Aubrey V., Hon Y., Shaw C., Burden S. (2019). Healthy Eating Interventions in Adults Living with and beyond Colorectal Cancer: A Systematic Review. J. Hum. Nutr. Diet..

[4] Aghajanpour M., Nazer M.R., Obeidavi Z., Akbari M., Ezati P., Kor M. (2017). Functional Foods and Their Role in Cancer Prevention and Health Promotion: A Comprehensive Review. Am. J. Cancer Res..

[5] Liu S., Lee I.-M., Ajani U., Cole S.R., Buring J.E., Manson J.E. (2001). Intake of Vegetables Rich in Carotenoids and Risk of Coronary Heart Disease in Men: The Physicians’ Health Study. Int. J. Epidemiol..

[6] Schwingshackl L., Bogensberger B., Hoffmann G. (2018). Diet Quality as Assessed by the Healthy Eating Index, Alternate Healthy Eating Index, Dietary Approaches to Stop Hypertension Score, and Health Outcomes: An Updated Systematic Review and Meta-Analysis of Cohort Studies. J. Acad. Nutr. Diet..

[7] Müller M.J., Koertzinger I., Mast M., Langnäse K., Grund A. (1999). Physical Activity and Diet in 5 to 7 Years Old Children. Public Health Nutr..

[8] Torres S.J., Nowson C. (2007). Relationship between Stress, Eating Behaviour and Obesity. Nutrition.

[9] Berkman N.D., Lohr K.N., Bulik C.M. (2007). Outcomes of Eating Disorders: A Systematic Review of the Literature. Int. J. Eat. Disord..

[10] O’Brien K.M., Whelan D.R., Sandler D.P., Hall J.E., Weinberg C.R. (2017). Predictors and Long-Term Health Outcomes of Eating Disorders. PLoS ONE.

[11] Ahrberg M., Trojca D., Nasrawi N., Vocks S. (2011). Body Image Disturbance in Binge Eating Disorder: A Review. Eur. Eat. Disord. Rev..

[12] Rimm E.B., Giovannucci E.L., Stampfer M.J., Colditz G.A., Litin L.B., Willett W.C. (1992). Reproducibility and Validity of an Expanded Self-Administered Semiquantitative Food Frequency Questionnaire among Male Health Professionals. Am. J. Epidemiol..

[13] Willett W.C., Sampson L., Stampfer M.J., Rosner B., Bain C., Witschi J., Hennekens C.H., Speizer F.E. (1985). Reproducibility and Validity of a Semiquantitative Food Frequency Questionnaire. Am. J. Epidemiol..

[14] Kristal A.R., Peters U., Potter D.P. (2005). Is It Time to Abandon the Food Frequency Questionnaire?. Cancer Epidemiol. Biomarkers Prev..

[15] Arbit N., Ruby M., Rozin P. (2017). Development and Validation of the Meaning of Food in Life Questionnaire (MFLQ): Evidence for a New Construct to Explain Eating Behavior. Food Qual. Prefer..

[16] Framson C., Kristal A.R., Schenk J.M., Littman A.J., Zeliadt S., Benitez D. (2009). Development and Validation of the Mindful Eating Questionnaire. J. Am. Diet. Assoc..

[17] Sproesser G., Klusmann V., Schupp H.T., Renner B. (2017). Self-Other Differences in Perceiving Why People Eat What They Eat. Front. Psychol..

[18] Van Strien T., Frijters J.E.R., Bergers G.P.A., Defares P.B. (1986). The Dutch Eating Behavior Questionnaire (DEBQ) for Assessment of Restrained, Emotional, and External Eating Behavior. Int. J. Eat. Disord..

[19] Fairburn C.G., Beglin S.J. (1994). Assessment of Eating Disorders: Interview or Self-Report Questionnaire?. Int. J. Eat. Disord..

[20] Garner D.M. (1991). EDI-2: Eating Disorder Inventory-2.

[21] Luce K.H., Crowther J.H. (1999). The Reliability of the Eating Disorder Examination—Self-report Questionnaire Version (EDE-Q). Int. J. Eat. Disord..

[22] Berg K.C., Peterson C.B., Frazier P., Crow S.J. (2012). Psychometric Evaluation of the Eating Disorder Examination and Eating Disorder Examination-Questionnaire: A Systematic Review of the Literature. Int. J. Eat. Disord..

[23] Guenther P.M., Casavale K.O., Reedy J., Kirkpatrick S.I., Hiza H.A.B., Kuczynski K.J., Kahle L.L., Krebs-Smith S.M. (2013). Update of the Healthy Eating Index: HEI-2010. J. Acad. Nutr. Diet..

[24] Krebs-Smith S.M., Pannucci T.E., Subar A.F., Kirkpatrick S.I., Lerman J.L., Tooze J.A., Wilson M.M., Reedy J. (2018). Update of the Healthy Eating Index: HEI-2015. J. Acad. Nutr. Diet..

[25] Peltner J., Thiele S. (2017). Association between the Healthy Eating Index-2010 and Nutrient and Energy Densities of German Households’ Food Purchases. Eur. J. Public Health.

[26] Thompson F.E., Subar A.F. (2017). Dietary Assessment Methodology. Nutrition in the Prevention and Treatment of Disease.

[27] Martin-Moreno J.M., Boyle P., Gorgojo L., Maisonneuve P., Fernandez-Rodriguez J.C., Salvini S., Willett W.C. (1993). Development and Validation of a Food Frequency Questionnaire in Spain. Int. J. Epidemiol..

[28] Turconi G., Celsa M., Rezzani C., Biino G., Sartirana M.A., Roggi C. (2003). Reliability of a Dietary Questionnaire on Food Habits, Eating Behaviour and Nutritional Knowledge of Adolescents. Eur. J. Clin. Nutr..

[29] Steptoe A., Pollard T.M., Wardle J. (1995). Development of a Measure of the Motives Underlying the Selection of Food: The Food Choice Questionnaire. Appetite.

[30] Fuentes Artiles R., Staub K., Aldakak L., Eppenberger P., Rühli F., Bender N. (2019). Mindful Eating and Common Diet Programs Lower Body Weight Similarly: Systematic Review and Meta-analysis. Obes. Rev..

[31] Larson N.I., Perry C.L., Story M., Neumark-Sztainer D. (2006). Food Preparation by Young Adults Is Associated with Better Diet Quality. J. Am. Diet. Assoc..

[32] Olson K.L., Emery C.F. (2015). Mindfulness and Weight Loss: A Systematic Review. Psychosom. Med..

[33] Kristal A.R., Potter J.D. (2006). Not the Time to Abandon the Food Frequency Questionnaire: Counterpoint. Cancer Epidemiol. Biomarkers Prev..

[34] Laviolle B., Froger-Bompas C., Guillo P., Sevestre A., Letellier C., Pouchard M., Daubert J.-C., Paillard F. (2005). Relative Validity and Reproducibility of a 14-Item Semi-Quantitative Food Frequency Questionnaire for Cardiovascular Prevention. Eur. J. Prev. Cardiol..

[35] Gosadi I., Alatar A., Otayf M., AlJahani D., Ghabbani H., AlRajban W., Alrsheed A., Al-Nasser K. (2017). Development of a Saudi Food Frequency Questionnaire and Testing Its Reliability and Validity. Saudi Med. J..

[36] Schembre S., Greene G., Melanson K. (2009). Development and Validation of a Weight-Related Eating Questionnaire. Eat. Behav..

[37] Stunkard A.J., Messick S. (1985). The Three-Factor Eating Questionnaire to Measure Dietary Restraint, Disinhibition and Hunger. J. Psychosom. Res..

[38] Seale J.L. (2002). Predicting Total Energy Expenditure from Self-Reported Dietary Records and Physical Characteristics in Adult and Elderly Men and Women. Am. J. Clin. Nutr..

[39] Voss S., Kroke A., Klipstein-Grobusch K., Boeing H. (1997). Obesity as a Major Determinant of Underreporting in a Self-Administered Food Frequency Questionnaire: Results from the EPIC-Potsdam Study. Z. Für Ernährungswissenschaft.

[40] German Nutrition Society (2017). 10 Guidelines of the German Nutrition Society (DGE) for a Wholesome Diet.

[41] World Health Organization (2018). Healthy Diet—Fact Sheet No. 394 2018.

[42] World Health Organizaton (1998). Preparation and Use of Food-Based Dietary Guidelines.

[43] Hauner H., Bechthold A., Boeing H., Brönstrup A., Buyken A., Leschik-Bonnet E., Linseisen J., Schulze M., Strohm D., Wolfram G. (2012). Evidence-Based Guideline of the German Nutrition Society: Carbohydrate Intake and Prevention of Nutrition-Related Diseases. Ann. Nutr. Metab..

[44] Wolfram G., Bechthold A., Boeing H., Ellinger S., Hauner H., Kroke A., Leschik-Bonnet E., Linseisen J., Lorkowski S., Schulze M. (2015). Evidence-Based Guideline of the German Nutrition Society: Fat Intake and Prevention of Selected Nutrition-Related Diseases. Ann. Nutr. Metab..

[45] Osgood C.E. (1964). Semantic Differential Technique in the Comparative Study of Cultures1. Am. Anthropol..

[46] Campbell D.T., Fiske D.W. (1959). Convergent and Discriminant Validation by the Multitrait-Multimethod Matrix. Psychol. Bull..

[47] Farstad S.M., McGeown L.M., von Ranson K.M. (2016). Eating Disorders and Personality, 2004–2016: A Systematic Review and Meta-Analysis. Clin. Psychol. Rev..

[48] Dufresne L., Bussières E., Bédard A., Gingras N., Blanchette-Sarrasin A., Bégin PhD C. (2020). Personality Traits in Adolescents with Eating Disorder: A Meta-analytic Review. Int. J. Eat. Disord..

[49] Evers C., Dingemans A., Junghans A.F., Boevé A. (2018). Feeling Bad or Feeling Good, Does Emotion Affect Your Consumption of Food? A Meta-Analysis of the Experimental Evidence. Neurosci. Biobehav. Rev..

[50] Diehl J.M., Staufenbiel T. (1999). Inventar Zum Essverhalten Und Gewichtsproblemen (IEG).

[51] Dickson-Spillmann M., Siegrist M., Keller C. (2011). Development and Validation of a Short, Consumer-Oriented Nutrition Knowledge Questionnaire. Appetite.

[52] Beierlein C., Kovaleva A., Kemper C.J., Rammstedt B. (2014). Allgemeine Selbstwirksamkeit Kurzskala (ASKU). Zs. Soz. Items Skalen ZIS.

[53] Dalbert C., Montada L., Schmitt M. (1987). Glaube an Eine Gerechte Welt Als Motiv: Validierungskorrelate Zweier Skalen. Psychol. Beitrage.

[54] Beierlein C., Kovaleva A., László Z., Kemper C.J., Rammstedt B. (2015). Kurzskala zur Erfassung der Allgemeinen Lebenszufriedenheit (L-1). Zs. Soz. Items Skalen ZIS.

[55] Kodinariya T.M., Makwana P.R. (2013). Review on Determining Number of Cluster in K-Means Clustering. Int. J..

[56] R Core Team (2020). R: A Language and Environment for Statistical Computing. R Foundation for Statistical Computing.

[57] RStudio Team RStudio: Integrated Development for R. http://www.rstudio.com/.

[58] Revelle W. Psych: Procedures for Psychological, Psychometric, and Personality Research. https://CRAN.R-project.org/package=psych.

[59] Jorgensen T.D., Pornprasertmanit S., Schoemann A.M., Rosseel Y. SemTools: Useful Tools for Structural Equation Modeling. https://CRAN.R-project.org/package=semTools.

[60] Harrell F.E. Hmisc: Harrell Miscellaneous. https://cran.r-project.org/package=Hmisc.

[61] Kassambara A., Mundt F. Package ‘Factoextra’. https://cran.microsoft.com/snapshot/2016-11-30/web/packages/factoextra/factoextra.pdf.

[62] Hemmelmann C., Brose S., Vens M., Hebebrand J., Ziegler A. (2010). Perzentilen des Body-Mass-Index auch für 18- bis 80-Jährige? Daten der Nationalen Verzehrsstudie II. DMW Dtsch. Med. Wochenschr..

[63] Grad F.P. (2002). The Preamble of the Constitution of the World Health Organization. Bull. World Health Organ..

[64] Ritchie H., Roser M. Meat and Dairy Production. https://ourworldindata.org/meat-production.

[65] Abbasi F., Brown B.W., Lamendola C., McLaughlin T., Reaven G.M. (2002). Relationship between Obesity, Insulin Resistance, and Coronary Heart Disease Risk. J. Am. Coll. Cardiol..

[66] Stommel M., Schoenborn C.A. (2010). Variations in BMI and Prevalence of Health Risks in Diverse Racial and Ethnic Populations. Obesity.

[67] Aballay L.R., Eynard A.R., Díaz M.D.P., Navarro A., Muñoz S.E. (2013). Overweight and Obesity: A Review of Their Relationship to Metabolic Syndrome, Cardiovascular Disease, and Cancer in South America. Nutr. Rev..

[68] Lavie C.J., Milani R.V., Ventura H.O. (2009). Obesity and Cardiovascular Disease. J. Am. Coll. Cardiol..

[69] Greeno C.G., Jackson C., Williams E.L., Fortmann S.P. (1998). The Effect of Perceived Control over Eating on the Life Satisfaction of Women and Men: Results from a Community Sample. Int. J. Eat. Disord..

[70] He J., Zhao Y., Zhang H., Lin Z. (2020). Orthorexia Nervosa Is Associated with Positive Body Image and Life Satisfaction in Chinese Elderly: Evidence for a Positive Psychology Perspective. Int. J. Eat. Disord..

[71] Matthews M., Zullig K.J., Ward R.M., Horn T., Huebner E.S. (2012). An Analysis of Specific Life Satisfaction Domains and Disordered Eating among College Students. Soc. Indic. Res..

[72] Leroy P., Requillart V., Soler L.G., Enderli G. (2016). An Assessment of the Potential Health Impacts of Food Reformulation. Eur. J. Clin. Nutr..

[73] Salles C., Kerjean J.R., Veiseth-Kent E., Stieger M., Wilde P., Cotillon C., Consortium T. (2017). The TeRiFiQ Project: Combining Technologies to Achieve Significant Binary Reductions in Sodium, Fat and Sugar Content in Everyday Foods Whilst Optimising Their Nutritional Quality. Nutr. Bull..

[74] Belc N., Smeu I., Macri A., Vallauri D., Flynn K. (2019). Reformulating Foods to Meet Current Scientific Knowledge about Salt, Sugar and Fats. Trends Food Sci. Technol..

[75] Feskens E.J.M., Frewer L.J., Gregory P., Grusak M., Parnell W., Scholten M.C.T., Wenink J., McKhann H., Byrne P., Gøtke N. FACCE JPI-JPI HDHL Priority Joint Actions to Contribute to the European Strategy on Food and Nutrition Security: Outcomes of the Grand Debate" Nutrition Security-A Whole System Approach". https://library.wur.nl/WebQuery/wurpubs/fulltext/407464.

[76] Brug J., van der Ploeg H.P., Loyen A., Ahrens W., Allais O., Andersen L.F., Cardon G., Capranica L., Chastin S., De Bourdeaudhuij I. (2017). Determinants of Diet and Physical Activity (DEDIPAC): A Summary of Findings. Int. J. Behav. Nutr. Phys. Act..

[77] Rousseeuw P.J. (1987). Silhouettes: A Graphical Aid to the Interpretation and Validation of Cluster Analysis. J. Comput. Appl. Math..

[78] Ocke M. (1997). The Dutch EPIC Food Frequency Questionnaire. I. Description of the Questionnaire, and Relative Validity and Reproducibility for Food Groups. Int. J. Epidemiol..

[79] Paalanen L., Männistö S., Virtanen M.J., Knekt P., Räsänen L., Montonen J., Pietinen P. (2006). Validity of a Food Frequency Questionnaire Varied by Age and Body Mass Index. J. Clin. Epidemiol..

[80] Gardner M.P., Wansink B., Kim J., Park S.-B. (2014). Better Moods for Better Eating?: How Mood Influences Food Choice. J. Consum. Psychol..

[81] Baucom D.H., Aiken P.A. (1981). Effect of Depressed Mood on Eating among Obese and Nonobese Dieting and Nondieting Persons. J. Pers. Soc. Psychol..

[82] Martin C.K., Rosenbaum D., Han H., Geiselman P.J., Wyatt H.R., Hill J.O., Brill C., Bailer B., Miller-III B.V., Stein R. (2011). Change in Food Cravings, Food Preferences, and Appetite During a Low-Carbohydrate and Low-Fat Diet. Obesity.

[83] Gero D., Steinert R.E., le Roux C.W., Bueter M. (2017). Do Food Preferences Change After Bariatric Surgery?. Curr. Atheroscler. Rep..

[84] Flaudias V., Iceta S., Zerhouni O., Rodgers R.F., Billieux J., Llorca P.-M., Boudesseul J., De Chazeron I., Romo L., Maurage P. (2020). COVID-19 Pandemic Lockdown and Problematic Eating Behaviors in a Student Population. J. Behav. Addict..

[85] Di Renzo L., Gualtieri P., Pivari F., Soldati L., Attinà A., Cinelli G., Leggeri C., Caparello G., Barrea L., Scerbo F. (2020). Eating Habits and Lifestyle Changes during COVID-19 Lockdown: An Italian Survey. J. Transl. Med..

[86] Chao A., Thun M.J., Connell C.J., McCullough M.L., Jacobs E.J., Flanders W.D., Rodriguez C., Sinha R., Calle E.E. (2005). Meat Consumption and Risk of Colorectal Cancer. JAMA.

[87] Johnston B.C., Zeraatkar D., Han M.A., Vernooij R.W., Valli C., El Dib R., Marshall C., Stover P.J., Fairweather-Taitt S., Wójcik G. (2019). Unprocessed Red Meat and Processed Meat Consumption: Dietary Guideline Recommendations from the Nutritional Recommendations (NutriRECS) Consortium. Ann. Intern. Med..

[88] McAfee A.J., McSorley E.M., Cuskelly G.J., Moss B.W., Wallace J.M., Bonham M.P., Fearon A.M. (2010). Red Meat Consumption: An Overview of the Risks and Benefits. Meat Sci..

