# Supplementary material for: Development and Validation of the General Dietary Behavior Inventory (GDBI) in Scope of International Nutrition Guidelines
Source: Nutrients. 2021 Apr 17;13(4):1328. doi: 10.3390/nu13041328 (PMC8073993; doi:10.3390/nu13041328)
Supplement: Supplementary file 1 [file nutrients-13-01328-s001.zip › nutrients-1138705-supplementary.pdf]

Supplementary Materials

Table S1. Original and translated items of the General Dietary Behavior Inventory

Scoring of each item: From 1 (=Like behavior A) to 5 (=Like behavior B)

Item code	Original item	Translated item
db1	Ich nehme täglich unterschiedliche Lebensmittel zu mir.<br><br>Im Vergleich zu<br><br>Ich nehme täglich die gleichen Lebensmittel zu mir.	I eat different foods every day.<br><br>Compared to<br><br>I eat the same foods every day.
db2	Meine Mahlzeiten beinhalten immer tierische Produkte (z.B. Fleisch, Fisch, Eier, Milchprodukte wie Joghurt, Sahne, Käse).<br><br>Im Vergleich zu<br><br>Meine Mahlzeiten beinhalten keine tierischen Produkte (z.B. Fleisch, Fisch, Eier, Milchprodukte wie Joghurt, Sahne, Käse).	My meals always include animal products (e.g., meat, fish, eggs, dairy products such as yogurt, cream, cheese).<br><br>Compared to<br><br>My meals do not include animal products (e.g., meat, fish, eggs, dairy products such as yogurt, cream, cheese).
db3	Meine Mahlzeiten beinhalten immer pflanzliche Produkte (z.B. Gemüse, Getreideprodukte, Tofuprodukte).<br><br>Im Vergleich zu<br><br>Meine Mahlzeiten beinhalten keine pflanzlichen Produkte (z.B. Gemüse, Getreideprodukte, Tofuprodukte).	My meals always include plant-based products (e.g., vegetables, grain products, tofu products).<br><br>Compared to<br><br>My meals do not include plant-based products (e.g., vegetables, grain products, tofu products).
db4	Ich esse täglich mindestens 2 Portionen Obst.<br>Im Vergleich zu<br>Ich esse nie Obst.	I eat at least 2 servings of fruit daily.<br><br>Compared to<br><br>I never eat fruit.

db5	Ich esse täglich mindestens 3 Portionen Gemüse.<br><br>Im Vergleich zu<br><br>Ich esse nie Gemüse.	I eat at least 3 servings of vegetables daily.<br><br>Compared to<br><br>I never eat vegetables.
db6	Ich esse täglich Vollkornprodukte (z.B. Vollkornnudeln, Vollkornbrot) anstatt Weißmehlprodukte (z.B. „herkömmliche“ Nudeln, Weißbrot/Graubrot).<br>Im Vergleich zu<br>Ich esse täglich Weißmehlprodukte (z.B. „herkömmliche“ Nudeln, Weißbrot/Graubrot) anstatt Vollkornprodukte (z.B. Vollkornnudeln, Vollkornbrot).	I eat whole grain products (e.g., whole grain pasta, whole grain bread) instead of white flour products (e.g., "conventional" pasta, white bread/grain bread) every day.<br><br>Compared to<br><br>I eat white flour products (e.g., "conventional" pasta, white bread/grain bread) instead of whole grain products (e.g., whole grain pasta, whole grain bread) every day.
db7	Ich nehme immer pflanzliche Fette (z.B Margarine und Öle auf Basis von Raps, Oliven, Sonnenblumen) anstatt tierischer Fette (Butter, Schmalz etc.) zu mir.<br><br>Im Vergleich zu<br><br>Ich nehme immer tierische Fette (Butter, Schmalz etc.) anstatt pflanzlicher Fette (z.B. Margarine und Öle auf Basis von Oliven, Raps, Sonnenblumen) zu mir.	I always consume vegetable fats (e.g., margarine and oils based on canola, olive, sunflower) instead of animal fats (butter, lard, etc.).<br><br>Compared to<br><br>I always consume animal fats (butter, lard, etc.) instead of vegetable fats (e.g., margarine and oils based on olives, canola, sunflowers).
db8	Ich esse keine Süßigkeiten (z.B. Schokolade, Kekse, Gebäck).<br><br>Im Vergleich zu<br><br>Ich esse täglich Süßigkeiten (z.B. Schokolade, Kekse, Gebäck).	I do not eat sweets (e.g. chocolate, cookies, pastries).<br><br>Compared to<br><br>I eat sweets (e.g., chocolate, cookies, pastries) every day.
db9	Ich esse keine Fast-Food- und Fertigprodukte (z.B. Tiefkühlpizza, Mikrowellenfertiggerichte).<br><br>Im Vergleich zu	I do not eat fast food and convenience foods (e.g., frozen pizza, microwave ready-to-eat meals).<br><br>Compared to

	Ich esse täglich Fast-Food- und Fertigprodukte (z.B. Tiefkühlpizza, Mikrowellenfertiggerichte).	I eat fast food and convenience foods (e.g., frozen pizza, microwave ready-to-eat meals) daily.
db10	Ich trinke keine zucker-/ süßungsmittelhaltige Getränke (z.B. Säfte, Limonade, gesüßter Kaffee/Tee).<br><br>Im Vergleich zu<br><br>Ich trinke täglich zucker-/ süßungsmittelhaltige Getränke (z.B. Säfte, Limonade, gesüßter Kaffee/Tee).	I do not drink sugary/sweetened beverages (e.g., juices, soda, sweetened coffee/tea).<br><br>Compared to<br><br>I drink sugary/sweetened beverages (e.g., juices, lemonade, sweetened coffee/tea) daily.
db11	Ich nehme täglich mindestens 1,5 Liter Flüssigkeit (ausgenommen alkoholische Getränke bzw. zucker-/süßungsmittelhaltige Getränke) zu mir.<br><br>Im Vergleich zu<br><br>Ich nehme täglich weniger als 1,5 Liter Flüssigkeit (ausgenommen alkoholische Getränke bzw. zucker-/süßungsmittelhaltige Getränke) zu mir.	I consume at least 1.5 liters of fluid daily (excluding alcoholic beverages or beverages containing sugar/sweeteners).<br><br>Compared to<br><br>I consume less than 1.5 liters of fluid (excluding alcoholic beverages or beverages containing sugar/sweeteners) daily.
r_db12	Bei der Zubereitung der Mahlzeiten ist mir wichtig, dass diese schnell zubereitet werden (z.B. schnelles Frittieren, schnelles/scharfes Anbraten).<br><br>Im Vergleich zu<br><br>Bei der Zubereitung der Mahlzeiten ist mir wichtig, dass diese langsam zubereitet werden (z.B. langsames Garen/Braten mit mittlerer Hitze).	When preparing meals, it is important to me that they are prepared quickly (e.g., quick frying, quick/spicy sautéing).<br><br>Compared to<br><br>When preparing meals, it is important to me that they are prepared slowly (e.g., slow cooking/frying with medium heat).
db13	Beim Würzen meiner Speisen achte ich darauf, möglichst wenig Salz einzusetzen.<br><br>Im Vergleich zu<br><br>Beim Würzen der Speisen nutze ich gerne viel Salz.	When seasoning my meals, I make sure to use as little salt as possible.<br><br>Compared to<br><br>I like to use a lot of salt when seasoning my food.

r_db14	Ich trinke regelmäßig (täglich oder auch jedes Wochenende) Alkohol.<br><br>Im Vergleich zu<br><br>Ich trinke keinen oder sehr selten (nur zu besonderen Anlässen) Alkohol.	I drink alcohol regularly (daily or even every weekend).<br><br>Compared to<br><br>I don't drink alcohol or drink it very rarely (only on special occasions).
db15	Ich esse lieber in Ruhe, ohne dass ich von irgendetwas abgelenkt werde.<br><br>Im Vergleich zu<br><br>Während des Essens beschäftige ich mich gerne mit anderen Themen (z.B. Fernsehen, Gedanken an die Arbeit, Lesen).	I prefer to eat in peace without being distracted by anything.<br><br>Compared to<br><br>While eating, I like to occupy myself with other topics (e.g., watching TV, thinking about work, reading).
db16	Bei der Einnahme meiner Mahlzeiten lasse ich mir gerne Zeit.<br><br>Im Vergleich zu<br><br>Meine Mahlzeiten nehme ich gerne schnell ein.	When taking my meals, I like to take my time.<br><br>Compared to<br><br>I like to take my meals quickly.

Table 2. Intercorrelations of General Dietary Behvior Inventory items.

	db1	db2	db3	db4	db5	db6	db7	db8	db9	db10	db11	db12	db13	db14	db15	db16
db1																
db2	0.07															
db3	0.26***	0.18***														
db4	0.12*	0.22***	0.30***													
db5	0.29***	0.28***	0.50***	0.43***												
db6	0.09	0.28***	0.33***	0.27***	0.37***											
db7	0.13**	0.16***	0.34***	0.22***	0.31***	0.30***										
db8	-0.04	0.15**	0.10*	0.07	0.15**	0.16***	0.03									
db9	0.16***	0.22***	0.22***	0.26***	0.23***	0.24***	0.12*	0.28***								
db10	0.07	0.20***	0.17***	0.19***	0.17***	0.31***	0.11*	0.27***	0.35***							
db11	0.10*	0.22***	0.14**	0.23***	0.26***	0.26***	0.07	0.10*	0.24***	0.37***						
db12	0.30***	0.06	0.14**	0.19***	0.17***	0.16***	0.07	0.10*	0.31***	0.15**	0.12*					
db13	0.07	0.06	-0.01	0.11*	0.08	0.22***	0.11*	0.09	0.13**	0.13**	0.09	0.23***				
db14	-0.03	-0.10*	0.05	0.11*	0.08	0.08	0.03	0.01	0.04	-0.00	-0.05	0.11*	0.19***			
db15	0.20***	0.18***	0.07	0.12*	0.11*	0.19***	-0.04	0.13**	0.16**	0.06	0.12*	0.25***	0.17***	0.06		
db16	0.16***	0.08	0.14**	0.07	0.16**	0.08	0.07	0.08	0.17***	-0.05	0.03	0.26***	0.12*	0.08	0.39***	

* $p \leq .05$, ** $p < .01$, *** $p < .001$
